# Healthy aging of the left ventricle in relationship to cardiovascular risk factors: The Multi-Ethnic Study of Atherosclerosis (MESA)

**DOI:** 10.1371/journal.pone.0179947

**Published:** 2017-06-22

**Authors:** Chia-Ying Liu, Shenghan Lai, Nadine Kawel-Boehm, Harjit Chahal, Bharath Ambale-Venkatesh, Joao A. C. Lima, David A. Bluemke

**Affiliations:** 1 Radiology and Imaging Sciences, National Institutes of Health (NIH), Bethesda, Maryland, United States of America; 2 Department of Pathology, Johns Hopkins School of Medicine, Baltimore, Maryland, United States of America; 3 Kantonsspital Graubuenden, Clinic of Radiology, Chur, Switzerland; 4 Department of Radiology, Johns Hopkins Hospital, Baltimore, Maryland, United States of America; Medical University Innsbruck, AUSTRIA

## Abstract

**Background:**

Understanding the relationship of cardiovascular structure and function to age is confounded by the high prevalence of traditional risk factors in the United States. The purpose of the study is to compare left ventricular (LV) and aortic structural, and functional parameters in individuals with and without traditional risk factors in a population-based cohort.

**Methods and results:**

3015 study participants (48% men, age 55–94, mean 69.01±9.17 years) in the Multi-Ethnic Study of Atherosclerosis (MESA) underwent cardiovascular magnetic resonance (CMR) imaging from 2010–2012. Absence of cardiovascular (CV) risk factors (no hypertension, diabetes or impaired fasting glucose, obesity, smoking or hypercholesterolemia) was infrequent, occurring in just 314 (10.4%, 38% men) of 3015 participants. In multivariable analyses adjusting for age, sex and race, individuals with CV risk factors had significantly larger LV mass index (by 17%) and lower LV contractibility (circumference strain, lower by 14%). Indexed LV volumes and stroke volume were inversely associated with age, but such relationships were not statistically significant in risk-free male subjects (p>0.05). Men with CV risk factors showed positive association of CMR T1 indices of myocardial fibrosis with age. Aortic function was similar in individuals with and without risk factors; age was associated with decline of aortic function in both CV and no CV risk factor groups.

**Conclusion:**

Our results support that LV structure and function are better preserved in senescent hearts in the absence of traditional cardiovascular risk factors, and such protection is more prominent in men than in women.

## Introduction

Advanced age is associated with changes at the level of the individual myocyte [1, 2] including biochemical alterations at the subcellular level, [3–5] cardiomyocyte loss and hypertrophy, changes in collagen type and degree of cross-linking. These molecular and cellular processes are believed to contribute to ascertainable changes in left ventricular (LV) morphology and function that characterize the aging process. There is an increasing evidence-based relationship between aging and reduction in LV cavity size out of proportion to LV mass, increase in LV wall thickness, and elevation or no change in global LV systolic function. Subclinical reduction in regional systolic and diastolic function is also reported [6–8]. Some of the prominent aspects of this age-related remodeling have been independently linked to adverse clinical consequences [9].

Traditional cardiovascular risk factors such as obesity, smoking, diabetes, hypertension, and dyslipidemia are known to accelerate ventricular and vascular aging and either directly or indirectly alter LV mass, size, and systolic function [10–12]. Furthermore, their prevalence and extent tends to increase with population aging, thus potentially confounding the independent effects of age in relationship to cardiovascular structure and function. Previous studies that have evaluated the effects of aging on cardiac remodeling have not meticulously excluded subjects with known cardiovascular risk factors. Thus the separate effects and magnitude of advanced age independent of risk factors on cardiovascular structure and function remains unclear.

The Multi-Ethnic Study of Atherosclerosis (MESA) examination cohort consists of community-dwelling men and women who had no history of clinical cardiovascular disease at the baseline. Since cardiovascular risk factors are extremely common in the United States, for convenience, we describe individuals without such risk factors as “risk-free”. By characterizing the cardiovascular phenotype of this risk-free population, cardiac function parameters relevant to healthy aging might also be defined to help ascertain risk factor impact on cardiac structure and function.

## Methods

### MESA cohort

The Multi-Ethnic Study of Atherosclerosis (MESA) is a multicenter prospective cohort study to explore the prevalence, correlation and progression of subclinical cardiovascular disease in Caucasians, African-Americans, Hispanics, and Chinese. Details regarding to the overall design, recruitment, and methods of the MESA study have been described previously [13]. The cohort was selected from six US communities (Baltimore city and Baltimore county, Maryland; Chicago, Illinois; Northern Manhattan, New York, Forsyth County, North Carolina; Los Angeles County, California; and St. Paul, Minnesota) who had no history of clinical cardiovascular disease at baseline (year 2000) and excluded those undergoing treatment for cancer, pregnancy, weight over 300 pounds, a chest CT scan in the prior year, cognitive inability or language barrier (other than English, Spanish, Cantonese or Mandarin), and conditions impeding long term follow up (serious medical condition, living in a nursing home, or plans to leave the community within five years). Examination 5 of the MESA study (year 2010), after approximately 10 years of follow-up of the cohort, consisted of 4655 participants (68.5±8.8 years, range 54–94 years), 3015 (64%) of whom agreed to undergo cardiovascular magnetic resonance (CMR) imaging of the heart and aorta. The institutional review board at all participating centers and NHLBI approved the study (Columbia University Medical Center Institutional Review Board, the Johns Hopkins University School of Medicine Joint Committee on Clinical Investigation, the University of Minnesota Human Research Protection Program, the Northwestern University Social and Behavioral Sciences Institutional Review Board, the Harbor-University of California Los Angeles [UCLA] Research and Education Institute Human Subjects Committee, the UCLA Office of Human Research Protection Program, the University of Vermont Committees on Human Research, the Wake Forest University Health Sciences Office of Research Institutional Review Board, and the University of Washington Human Subjects Division), and all participants gave informed consent.

### Definition of risk-free (n = 314 subjects in MESA 5)

“Risk-free” participants were defined as study subjects without any of the following cardiovascular risk factors at MESA exanimation 5: body mass index (BMI)≥30, former/current smokers, hypertension diagnosis or systolic blood pressure>140 mmHg and diastolic blood pressure>90, impaired fasting glucose or diabetes, total cholesterol≥240 mg/dl, LDL cholesterol≥160 mg/dl, HDL cholesterol<40 mg/dl, and triglycerides≥150 mg/dl.

### Magnetic resonance imaging

The CMR protocol was that of the fifth examination of MESA. All participants were imaged using 1.5T whole-body MRI systems (Signa LX, GE Healthcare, Waukesha, Wisconsin; Avanto or Espree, Siemens Medical Systems, Erlangen, Germany) with a phased-array coil for signal reception placed around the thorax with anterior and posterior elements selected around the heart. Participants were instructed to hold their breath at functional residual capacity during imaging. To measure ventricular function, the entire heart was imaged in long and short-axis orientations with cine steady state free precession (SSFP) sequences using CIM software (version 6.2, Auckland MRI Research Group, University of Auckland, New Zealand). All cine images had an acquired temporal resolution of <40 ms, retrospectively reconstructed as 50 cine frames at 20–35 ms intervals over the cardiac cycle. Left ventricular heart function including myocardial mass, volume, stroke volume, and ejection fraction was measured through contouring of the left ventricle. To assess the regional myocardial contractility, myocardial MR-tagged images were obtained in the short-axis plane at the basal, mid-LV and apical levels and analyzed using Harmonic Phase software (Diagnosoft, Palo Altro, CA). Gadolinium contrast (0.15mmol/kg, Magnevist; Bayer Healthcare Pharmaceuticals, New Jersey, USA) was used for the assessment of fibrosis. Late gadolinium enhancement (LGE) images were obtained at 15 minutes after injection and analyzed using QMass (version 7.2; Medis, Leiden, the Netherlands). Myocardial T1 values were obtained by modified Look-Locker inversion recovery (MOLLI) sequence [14, 15]. One mid-ventricular short-axis slice was acquired before and after gadolinium contrast injections at 12 and 25 minutes. T_1_ maps were constructed offline using MASS research software (Department of Radiology, Leiden University Medical Center, Leiden, The Netherlands).

Phase contrast cine gradient echo sequence was also obtained to evaluate aortic stiffness including ascending and descending aortic distensibility (AAD and DAD), and pulse wave velocity (PWV) [16]. An aortic sagittal oblique plane with a black-blood sequence was acquired to position the aortic phase-contrast imaging, and also allowed for the measurement of the path-length between the ascending and descending aorta to calculate aortic PWV. Aortic function was measured with an automated contour detection software (ARTFUN, INSERM LIM).

### Statistical analyses

Age and LV/aortic structural and functional indices were treated as continuous variables. LV parameters were divided by body surface area (BSA) (kg/m^2^) according to DuBois and DuBois formula. The following LV and aortic measurements were calculated (mean±SD) in each group: i) Mass ii) End-diastolic volume (EDV) iii) End-systolic volume (ESV) iv) Ejection fraction (EF) v) Mass to end-diastolic volume (M/V) ratio vi) Stroke volume (SV) vii) Peak regional systolic circumferential strain (Ecc) in 12 segments viii) T1 indices including extracellular volume fraction (ECV), partition coefficient, pre and post-contrast T1 values. ix) Aortic stiffness including AAD, DAD, and PWV. Differences in the demographic characteristics and LV/aortic indices between the risk-free (i.e., no hypertension, diabetes or impaired fasting glucose, obesity, smoking or hypercholesterolemia) and the remaining MESA population (with-risk) were studied using unpaired t-test for continuous variables and Chi-square test for categorical variables. Comparison of the LV indices (absolute and indexed by BSA), Ecc, T1 indices, and aortic function between groups was adjusted for age, sex, and race. Ecc in 12 LV segments (anterior, lateral, inferior and septal) from the basal, mid, and apical slices were compared between groups. To account for the correlation among the 12 regional strains (Ecc), which were obtained from the same person, a generalized estimating (GEE) model was used to examine whether being supernormal is associated with regional left ventricular function [17]. Percentages of participants with positive LGE from the exam5 MRI study and with any CVD events were also compared between the risk-free and with-risk groups. CVD events included myocardial infarction, resuscitated cardiac arrest, definite angina, probable angina (if followed by revascularization), and stroke that were reported between the baseline and December 31^st^, 2012. We further listed LV mass index and M/V ratio of both risk-free and with-risk groups stratified by gender and race/ethnicity, and examine the difference between Black and White of each category using unpaired t-test.

Age dependence of each CMR measurement was evaluated using univariate linear regression stratified 1) by gender 2) by race/ethnicity in both groups. To explore the association between each indexed LV and aortic indices and risk factors, linear regression was performed with multivariable adjustment for demographic characteristics (age, sex, race) and risk factors (categorized factors that defined the risk-free, except HDL because it is closely related to total cholesterol and LDL) in the entire cohort without dividing into groups. All analyses were performed using SAS and significance was declared as p<0.05.

## Results

The study sample included 3015 participants with complete cardiac global function measurements including LV mass, volume, stroke volume, and ejection fraction. The mean age was 69.01±9.17 years. In the entire cohort, 48% were men. The race/ethnic distribution of the full cohort was 42.2% Non-Hispanic white, 24.8% African-American, 20.4% Hispanic and 12.6% Chinese-American.

**Fig 1:**
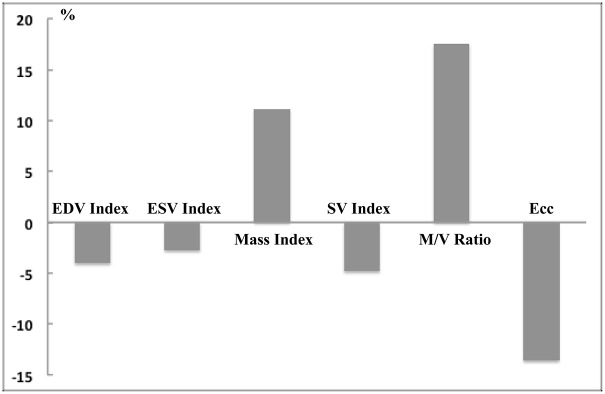
Comparison of LV indices between study subjects with versus without cardiovascular risk factors. Percent difference [100×(with-risk−risk-free) ⁄ risk-free] are shown. All displayed indices are significantly different between with-risk and risk-free groups.

Three hundred fourteen subjects of 3015 subjects (10.4%) had no traditional risk factors (‘risk-free’) (Table 1). Participants with risk-free status were approximately 3 years younger and more likely to be female. Study subjects with risk factors had lower indexed LV volume (by approximately 4% for EDV and 3% for ESV) and larger indexed mass (by approximately 11%); as a result, the with-risk participants had 17% higher mass-to-volume ratios than those participants without risks. Fig 1 plots the percent difference [100×(with-risk−risk-free) ⁄ risk- free] of the LV indices between groups. For LV and aortic function, Ecc, stroke volume index, and descending aortic distensibility, all values were significantly impaired in participants with risk compared to risk-free participants. Participants with risk also had lower post contrast T1 times compared with risk-free (455.6 vs 464.0 ms at 12 minutes, p<0.001). One hundred thirty four (134) study subjects with risk factors had myocardial scar by LGE CMR, while only six of the risk-free had myocardial scar (5.2% vs. 1.9%, respectively, p<0.01), and four out of six were non-ischemic scar with less than 2% of scar mass. Overall, 192 subjects had a history of CV event, 8 in the risk-free group (2.5%), and 184 in the group with risk factors (6.8%).

**Table 1:** Left-ventricular (LV) structure/function and aortic function of risk-free and with risk MESA participants. Values displayed as mean±SD (95% confidence interval) and * for p<0.05 between groups after multivariate adjustment for age/sex/race.

	**With risk** **N = 2701**	**Risk-free** **N = 314**
Age (years)	69.3±9.2(69;69.6)	66.5±8.8(65.5;67.5)
Male (%)	49%	38%
EDV (ml)	119.6±32(118.3;120.7)	115.9±29.2(113.4;120.1)
ESV (ml)	46.3±18.5(45.6;47)	44.3±14.8(43.1;46.5)
Mass (g)	125.1±33.8(123.8;126.3)	104.7±28.4(102.3;108.8)*
SV (ml)	73.3±18.6(72.5;73.9)	71.6±17.4(70;74)
EF (%)	61.9±7.4(61.6;62.2)	62.2±6.0(61.4;62.8)
M/V ratio (g/ml)	1.07±0.23(1.06;1.08)	0.91±0.17(0.9;0.93)*
EDV index (ml/m^2^)	64.2±14.1(63.6;64.9)	66.9±12.3(65.8;68.6)*
ESV index (ml/m^2^)	24.7±8.7(24.4;25)	25.4±6.8(24.9;26.4)*
Mass index (g/m^2^)	66.9±13.8(66.3;67.4)	60.2±11.6(59.2;61.9)*
SV index (ml/m^2^)	39.5±8.6(39.1;39.8)	41.5±7.9(40.7;42.5)*
Global Ecc (%)	-16.5±5.2(-16.7;-16.3)	-19.1±2.8(-19.4;-18.8)*
CVD events (%)	6.8%	2.5%*
	**N = 1877**	**N = 228**
PWV (m/sec)	9.0±4.3(8.8;9.2)	8.2±3.7(7.7;8.6)
	**N = 1556**	**N = 194**
AAD (%/mmHg)	1.57±1.09(1.51;1.63)	1.69±1.15(1.53;1.85)
DAD (%/mmHg)	1.98±1.28(1.92;2.05)	2.35±1.55(2.14;2.58)*
	**N = 1195**	**N = 140**
Native T1 (ms)	977.2±42.7(975;980)	976.1±40.9(967;981)
Post 12 min T1 (ms)	455.2±40.3(453;457)	463.5±38.9(461;473)*
Post 25 min T1 (ms)	518.6±41(516;520)	525.9±39(523;535)*
Partition coefficient	0.45±0.04(0.44;0.45)	0.45±0.03(0.44;0.46)
	**N = 559**	**N = 49**
ECV (%)	26.9±3(26.7;27.2)	27.5±2.7(26.2;27.8)

Risk-free: no hypertension, diabetes or impaired fasting glucose, obesity, smoking or hypercholesterolemia. LV structure index by BSA. EDV- End-diastolic volume. ESV- End-systolic volume. EF- Ejection fraction. M/V ratio- Mass to end-diastolic volume. SV- Stroke volume. Ecc- Peak regional systolic circumferential strain. CVD- Cardiovascular disease. PWV- Pulse wave velocity. ADD- Ascending aortic distensibility. DAD- Descending aortic distensibility. ECV- Extracellular volume fraction.

LV mass index and M/V ratio were further stratified by gender and race/ethnicity (Table 2). Blacks had the highest mean LV mass index and M/V ratio among all races. The differences were all significant when compared to those of White.

**Table 2:** Left-ventricular mass index by body surface area and mass to volume (M/V) ratio of risk-free and with risk MESA participants stratified by gender and race. Values displayed as mean±SD (95% confidence interval). * p<0.05 of the unpaired t-test between black and white in the corresponding category.

		Risk free		With risk	
		Women	Men	Women	Men
Mass index (g/m^2^)	White	N = 98 54±7.5(52.5;55.5)	N = 66 71.4±12(68.4;74.4)	N = 574 57.5±9(56.8;58.2)	N = 534 72.6±11.5(71.6;73.6)
	Chinese	N = 44 54.5±6.8(52.5;56.6)	N = 20 62.8±8.2(58.9;66.6)	N = 148 58±9(56.6;59.5)	N = 167 70.7±11.2(69;72.4)
	Black	N = 15 60.7±9.9(55.2;66.2)*	N = 15 71.9±13.7(64.3;79.5)	N = 410 62.3±10.9(61.3;63.4)*	N = 306 79.4±15.5(77.7;81.2)*
	Hispanic	N = 35 54.2±7.1(51.8;56.7)	N = 21 69.8±8.4(66;73.7)	N = 251 61.3±10(60;62.5)	N = 308 74.5±13.1(73;76)
M/V ratio (g/ml)	White	0.87±0.16(0.84;0.91)	0.97±0.19(0.93;1.02)	0.98±0.2(0.96;1)	1.12±0.26(1.09;1.14)
	Chinese	0.88±0.12(0.84;0.92)	0.96±0.16(0.89;1.04)	0.98±0.19(0.95;1.01)	1.07±0.19(1.04;1.09)
	Black	0.91±0.18(0.81;1.01)	0.99±0.15(0.9;1.07)	1.05±0.21(1.03;1.07)*	1.16±0.24(1.14;1.19)*
	Hispanic	0.86±0.11(0.82;0.89)	0.99±0.19(0.9;1.07)	1.02±0.19(1;1.05)	1.16±0.26(1.13;1.19)

### Cardiovascular structure and function and age

**Fig 2:**
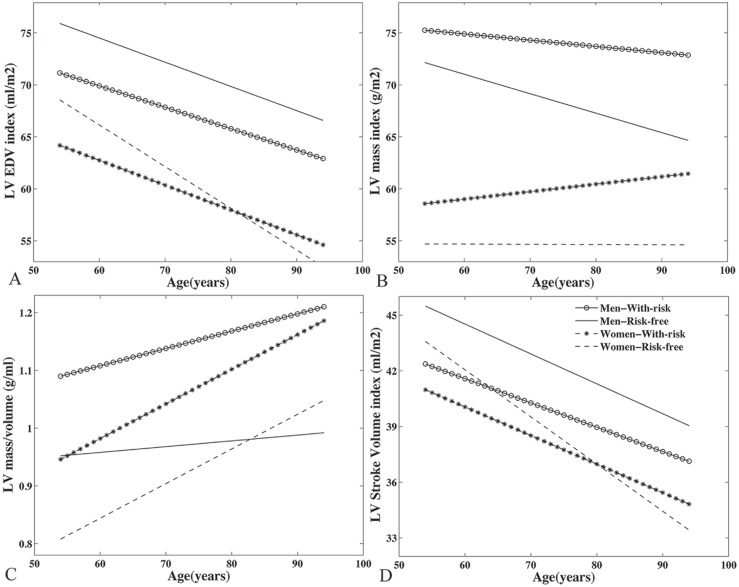
(A) LV EDV index is inversely associated with age in all groups. (B) LV mass index in women without risk factors was independent of age. M/V ratio was positively (C) and stroke volume index (D) was inversely associated with age but both changes are not significant in men without risk factors.

Table 3 displays the individual correlation coefficient (B) of each LV/aortic parameter, which represents change in the dependent variable per one-year increase in age, indicating that the effect of age varies according to risk status as well as sex. In general, LV volumes were inversely associated with age uniformly across groups (Fig 2A), but such relationships were not statistically significant in risk-free male subjects (p = 0.098 and 0.338 for EDV and ESV index respectively). Women with or without risk factors experienced overall similar age-related change in most of the LV indices. Risk-free women’s LV mass index was independent of age (p = 0.973), while women with risks had slightly but significant positive association with age (p = 0.014) in mass index (Fig 2B).

**Table 3:** The relationship of age with left-ventricular (LV) structure/function and aortic function in men and women with and without traditional risk factors. Univariate regression coefficient which represents change in the dependent variable per one-year increase in age. * for p<0.05.

	Women		Men	
	Risk-free	With risk	Risk-free	With risk
**LV Structure (N)**	192	1383	122	1315
EDV index (ml/m^2^)	-0.401*	-0.239*	-0.233	-0.206*
ESV index (ml/m^2^)	-0.147*	-0.085*	-0.072	-0.074*
Mass index (g/m^2^)	-0.002	0.072*	-0.187	-0.06
M/V ratio (g/ml)	0.006*	0.006*	0.001	0.003*
SV index (ml/m^2^)	-0.254*	-0.154*	-0.161	-0.131*
EF (%)	0.002	0.007	-0.01	-0.002
Global Ecc (%)	0.065*	0.01	0.000	-0.002
**LV Myocardium (N)**	84	555	56	640
Native T1 (ms)	-0.015	0.232	-0.085	0.863*
Post 12 min T1 (ms)	-0.865	0.008	-0.543	-0.543*
Post 25 min T1 (ms)	-1.466*	0.028	-0.681	-0.777*
Partition coefficient	0.001	0.000	0.001	0.001*
**N**	37	284	56	640
ECV (%)	0.031	0.029	-0.024	0.094*
**Aorta (N)**	125	786	69	757
PWV (m/sec)	0.169*	0.121*	0.174*	0.174*
AAD (%/mmHg)	-0.024*	-0.023*	-0.042*	-0.029*
DAD (%/mmHg)	-0.061*	-0.011*	-0.036*	-0.021*

LV structure index by body surface area. EDV- End-diastolic volume. ESV- End-systolic volume. EF- Ejection fraction. M/V ratio- Mass to end-diastolic volume. SV- Stroke volume. Ecc- Peak regional systolic circumferential strain. ADD- Ascending aortic distensibility. DAD- Descending aortic distensibility. PWV- Pulse wave velocity. ECV- Extracellular volume fraction.

Regardless of risk burden, LV mass index was not significantly associated with age in men. Age was not related to LV mass-to-volume (M/V) ratio in risk-free men (p = 0.503, Fig 2C). For LV function, SV was lower with age (Fig 2D, not significant for risk-free men) but both EF and Ecc were independent of age in all groups with the exception of Ecc that was slightly more positive (lower function) in risk-free women. For T1 measurements, significant age correlations were uniformly observed in men with risk factors. Age-associated deterioration in aortic functions (PWV, AAD and DAD) remained significant in both genders with or without risk factors.

When stratified by race/ethnicity (Table 4), Black had much less subjects (N = 30, 4% in all Black participants) in the “risk free” group. In the “with-risk” group, Blacks demonstrated the greatest age-dependent increases in ECV (beta = 0.088, p = 0.006) and native T1 (beta = 0.77, p = 0.008) among all ethnicities. Black was the only race showed age-dependent decrease in LV mass index regardless of risk status (Risk free: beta = -0.931, p = 0.003. With risk: beta = -0.128, p = 0.049).

**Table 4:** The relationship of age with left-ventricular (LV) structure/function and aortic function stratified by with and without traditional risk factors and race/ethnicity. Univariate regression coefficient which represents change in the dependent variable per one-year increase in age. * for p<0.05.

	Without risk				With risk			
	White	Chinese	Black	Hispanic	White	Chinese	Black	Hispanic
**LV Structure (N)**	164	64	30	56	1108	315	716	559
EDV index (ml/m^2^)	-0.355*	-0.171	-0.888*	-0.25	-0.253*	-0.12	-0.23*	-0.264*
ESV index (ml/m^2^)	-0.122*	-0.113	-0.215	-0.099	-0.087*	-0.045	-0.109*	-0.106*
Mass index (g/m^2^)	0.02	-0.036	-0.931*	-0.088	0.032	0.139	-0.128*	-0.035
M/V ratio (g/ml)	0.006*	0.003	0.000	0.002	0.005*	0.005*	0.002*	0.004*
SV index (ml/m^2^)	-0.232*	-0.058	-0.674*	-0.151	-0.166*	-0.074	-0.121*	-0.157*
EF (%)	0.000	0.056	-0.165	0.019	-0.002	0.018	0.034	0.017
Global Ecc (%)	0.044	-0.001	0.062	0.01	0.007	0.019	-0.013	-0.008
**LV Myocardium (N)**	84	27	13	16	609	121	295	170
Native T1 (ms)	-0.042	-0.855	1.331	-0.222	0.297	0.619	0.77*	0.664*
Post 12 min T1 (ms)	-0.424	-0.007	-2.019	0.481	-0.094	-0.53	-0.258	-0.121
Post 25 min T1 (ms)	-0.976*	-0.251	-2.797	0.385	-0.293	-0.234	-0.178	-0.449
Partition coefficient	0.001	0.000	0.000	0.000	0.001*	0.001	0.001*	0.000
**N**	43	0	3	3	332	0	150	77
ECV (%)	0.006	-	-0.057	0.176	0.047*	-	0.088*	0.066
**Aorta (N)**	97	56	16	25	654	245	410	234
PWV (m/sec)	0.109*	0.361*	0.123*	0.187*	0.12*	0.165*	0.156*	0.18*
AAD (%/mmHg)	-0.017	-0.06*	-0.013	-0.06*	-0.024*	-0.027*	-0.028*	-0.024*
DAD (%/mmHg)	-0.03*	-0.07*	-0.02	-0.164*	-0.015*	-0.031*	-0.021*	0.004

LV structure index by body surface area. EDV- End-diastolic volume. ESV- End-systolic volume. EF- Ejection fraction. M/V ratio- Mass to end-diastolic volume. SV- Stroke volume. Ecc- Peak regional systolic circumferential strain. ADD- Ascending aortic distensibility. DAD- Descending aortic distensibility. PWV- Pulse wave velocity. ECV- Extracellular volume fraction.

### Cardiovascular structure and function versus traditional risk factors

Table 5 summarizes the associations between the risk factors and each of the LV/aortic parameters of the entire cohort. In analyses adjusted for age, sex, and race, obesity (BMI≥30), hypertriglyceridemia (triglycerides≥150mg/dl), hypertension and diabetes were four leading risk factors that affected LV structure and function. Indexed LV volumes were smaller in diabetic subjects, but mass index and mass-to-volume ratios were greater than those of the non-diabetic hearts. Participants with myocardial scar had significantly larger LV volumes as well as higher mass than those without scar. Ejection fraction was directly related to hypertension and hypertriglyceridemia, but inversely related to smoking and the presence of myocardial scar. Cholesterol was mostly independent of these LV indices, except elevated LDL was associated with attenuated SV index.

**Table 5:** Multivariate analysis (adjusted for age, gender, race, and all of the risk factors in the table) of associations of left ventricular (LV) structure/function and aortic function versus risk factors (beta and * for p < 0.05).

	EDV index	ESV index	Mass index	SV index	EF	M/V ratio	Native T1	Post 12min T1	Post 25min T1	Partition coefficient	ECV	PWV	AAD	DAD
Hypertension	1.22*	0.06	5.05*	1.16*	0.76*	0.25*	0.038	-2.92	-2.02	-0.0019	0.13	0.2	-0.1	-0.2*
Diabetes	-3.03*	-1.24*	-0.67	-1.79*	0.11	0.15*	2.36	3.58	3.32	-0.0007	-0.21	-0.23	-0.08	-0.11
BMI ≥30	-2.69*	-0.94*	1.19*	-1.76*	-0.36	0.15*	2.84	-18*	-19.5*	-0.0049	-0.15	-0.29	0.1	-0.3*
Smokers	-0.61	0.26	0.88*	-0.88*	-0.66*	0.04	2.51	-1.06	-2.57	0.0047*	0.36	0.41*	-0.00	0.05
Total cholesterol ≥240mg/dl	0.2	-0.18	0.54	0.37	0.6	0.12	12.52	-1.76	-0.64	0.0031	-0.14	0.03	0.02	0.03
LDL ≥160 mg/dl	-2.28	-0.23	0.21	-2.05*	-1.12	-0.03	-7.51	-8.42	-7.23	0.0014	0.24	-0.39	0.09	-0.27
Triglycerides ≥150mg/dl	-3.41*	-2.1*	-1.2*	-1.31*	1.14*	0.2*	1.28	0.38	0.67	-0.0044	-0.75*	-0.05	0.08	0.16
Myocardial scar	5.1*	5.5*	7.78*	-0.41	-4.2*	-0.12	8.77*	-13.7*	-10.6*	0.0177*	-2.53	-0.29	-0.21	0.25

LV structure index by body surface area. EDV- End-diastolic volume. ESV- End-systolic volume. EF- Ejection fraction. M/V ratio- Mass to end-diastolic volume. SV- Stroke volume. ECV- Extracellular volume fraction. PWV- Pulse wave velocity. ADD- Ascending aortic distensibility. DAD- Descending aortic distensibility.

## Discussion

Given the increasing burden of cardiovascular risks in the aging US population, it is important to study the cumulative effects of risks in the cardiovascular structure and function due to the significant interactions between cardiovascular risk factors [18]. For the purpose of examining the effects of prevalent cardiovascular risk factors to LV pathophysiology, we divided the participants into two mutually exclusive groups: with and without traditional cardiovascular risk factors. Importantly, study subjects without risk factors had a very low incidence of CVD events (2.5%) and myocardial scar (1.9%). In this relatively large multi-ethnic CMR study, the major differences in LV structure and function between participants with or without risks were three fold: with-risk study subjects had greater mass index and smaller cavity volume index, had deteriorated LV contractility (defined by strain Ecc), and stroke volume index, and had greater age-associated LV remodeling.

In the Cardiovascular Health Study (CHS), a large cohort mainly Caucasians of 65 years and older was examined [11]. LV mass was significantly greater in participants with clinical coronary heart disease than those without. With the similar age distribution, our study extends their findings to a relatively healthy population with risk factors but without overt heart disease. In the Framingham Offspring Study [19] and the CARDIA study [20], lower LV mass was reported in both men and women with low risk burden compared to their high-risk counterparts.

The significance of age-related progression of LV measures depends on the risk status as well as sex and race. Compared to other age-dependent studies of LV structure and function, which mostly adjusted for pertinent risk factors, our results showed the collective impact because the interactions of risk factors might not be separable. Age association of LV mass has been controversial in the literatures. Previous longitudinal observations in Framingham Offspring Study by echocardiography have shown increase of LV mass over time in both men and women with or without risk burden [19]. Similar finding was also reported in the Baltimore Longitudinal Study on Aging with a wider range of age distribution (20–80 years) in healthy volunteers. However, using the CMR methods, Yeon et al. [21] showed LV mass (with or without indexation) decreased with advancing age in a subset of healthy cohort in Framingham Heart Study. Another study consisted of 390 normal volunteers using 3D echocardiography showed a borderline (p = 0.06) age association of LV mass index [22]. Our data allowed us to break down the perplex mass-age associations based on the risk status and sex as well as race. Normalization to BSA, LV mass did not show age dependence in all men and risk-free women but was positively associated to age in women with risks. In contrast to mass, smaller LV volumes with advancing age were consistently reported in the literature [6, 21–25]. However, we found that indexed LV volumes were independent of age in men without risks. These alterations led to the small but significant increase of M/V ratios in all groups but remained intact in the men without risk factors. Similar results in LV function also revealed that indexed stroke volume was preserved in healthy men while reduced significantly with age in the rest of groups. Previous MESA studies in the baseline [6] showed modestly increased EF with age, but no EF-age correlation existed in our current study. Of note, Ecc was slightly impaired in risk-free women, which was not detectable by ejection fraction. This demonstrates that tagging might be a more sensitive technique than cine. However, the mechanism underlying this correlation requires further investigation. When divided into race, Black had more LV/aorta indices with significant age dependency in the with-risk cohort and the greatest age-dependent decrease of stroke volume index (beta = -0.674, p = 0.002). Blacks also demonstrated the highest LV mass index and M/V ratio among all ethnicities. Many studies have suggested that Blacks compared with Whites have an increased prevalence of LV hypertrophy. Diagnosis of LV hypertrophy should be accompanied by ECG and clinical symptoms, which is beyond the scope of current study. However, our findings supported the previous study that Blacks have increased LV mass compared with Whites in the general populations.

For the T1 mapping analysis, we extended prior work [14] by dividing cohorts based on risk burdens and found that men in risks were more subjective to aging than the rest of groups as indicated in Table 2. Sado and co-workers [26] found no correlation between age and myocardial ECV in normotensive healthy volunteers, while in another study [27], weak but significant ECV increase with age was discovered in patients. Our findings were in part aligned with these studies and further suggested the men might be benefited more from keeping low risk burdens than women in preserving cardiac structure and function.

Traditional cardiovascular risk factors in relation to LV mass, volume and function have been reported in the MESA baseline study [10]. The present follow-up study comprised smaller but similar racial/ethnic groups. Although there was a difference in the cine CMR sequences to assess the LV parameters (gradient echo in the baseline versus SSFP in the follow-up) as well as the analysis methods, correlations between risk factors and LV measures were mostly consistent. In both studies among the traditional risk factors, hypertension was positively linked to normalized LV mass and EDV, stroke volume, and ejection fraction. Obesity was directly related to LV mass index but inversely related to LV volume index. This finding is consistent with previous reports of obesity as an independent risk factor for LV hypertrophy [28, 29]. Smoking was directly related to mass but inversely related to ejection fraction. One important factor that is worth noting is the triglycerides in which none of these associations persisted after multivariable adjustment in the MESA baseline analyses [10], but found to be a key factor in predicting the LV structure and function in the present follow-up study. Age was associated with a decline in aortic distensibility and with increased aortic stiffness as measured by PWV. This is also demonstrated by the little difference in the aortic PWV and distensibility between participants with and without cardiovascular risk burden (Table 1). This strongly suggests that arterial stiffness may be mainly driven by biological aging. Gender added to age remained as two dominate factors for all T1 mapping indices, which is in agreement with our previous analyses [14].

The major strength of the present study is an extensive presentation of LV/aortic indices in single MRI exanimation from a large community-based multi-ethnic cohort. The goal of our study is to examine the impact of risk factors as one entity. Hence, participants with events were included in the current analysis to represent better the overall burden of CVD in the US population. We have also analyzed the age associations (Table 2) excluding the participants with events but found only minimal variations in the correlation coefficients. The difference between two cohorts as presented in Table 1 was not altered, either. The limitations of this study include that our participants are mostly elderly and generalizability to other age groups is unknown. Risk factor definition and cut-off values for risk-free were identified based on prior cross-sectional study. Racial/ethnic was treated as covariate to maintain the statistical power.

In Summary, our results support that cardiac structure and function are preserved in senescent hearts without cardiovascular risk factors, and such protection is more prominent in men than in women. Additionally, obesity, hypertriglyceridemia, hypertension, and diabetes denote the major risks of LV structure and function. However, arterial stiffness might be unavoidable in biological aging.

## Abbreviations

ADD: Ascending aortic distensibility
BMI: Body mass index
BSA: Body surface area
CMR: Cardiovascular magnetic resonance
CVD: Cardiovascular disease
DAD: Descending aortic distensibility
ECV: Extracellular volume fraction
EDV: End-diastolic volume
EF: Ejection fraction
ESV: End-systolic volume
HDL: High-density lipoprotein
LDL: Low-density lipoprotein
LGE: Late gadolinium enhancement
LV: Left ventricular
M/V ratio: Mass to end-diastolic volume
MESA: Multi Ethnic Study of Atherosclerosis
PWV: Pulse wave velocity
SV: Stroke volume
